# Attachment, Personality and Locus of Control: Psychological Determinants of Risk Perception and Preventive Behaviors for COVID-19

**DOI:** 10.3389/fpsyg.2021.634012

**Published:** 2021-07-09

**Authors:** Sofia Tagini, Agostino Brugnera, Roberta Ferrucci, Ketti Mazzocco, Luca Pievani, Alberto Priori, Nicola Ticozzi, Angelo Compare, Vincenzo Silani, Gabriella Pravettoni, Barbara Poletti

**Affiliations:** ^1^Department of Neurology and Laboratory of Neuroscience, Istituto Auxologico Italiano, Istituto di Ricovero e Cura a Carattere Scientifico (IRCCS), Milan, Italy; ^2^Department of Human and Social Sciences, University of Bergamo, Bergamo, Italy; ^3^Department of Health Sciences, Aldo Ravelli Center for Neurotechnology and Experimental Brain Therapeutics, International Medical School, University of Milan, Milan, Italy; ^4^Neurology Clinic III, ASST Santi Paolo e Carlo, Milan, Italy; ^5^Istituto di Ricovero e Cura a Carattere Scientifico (IRCCS) Ca' Granda Foundation Maggiore Policlinico Hospital, Milan, Italy; ^6^Department of Oncology and Hemato-Oncology, University of Milan, Milan, Italy; ^7^European Institute of Oncology, IRCCS, Milan, Italy; ^8^Department of Pathophysiology and Transplantation, Dino Ferrari Center, University of Milan, Milan, Italy

**Keywords:** COVID-19, risk perception, preventive behaviors, psychological determinants, pandemic management

## Abstract

**Background:** The understanding of factors that shape risk perception is crucial to modulate the perceived threat and, in turn, to promote optimal engagement in preventive actions.

**Methods:** An on-line, cross-sectional, survey was conducted in Italy between May and July 2020 to investigate risk perception for COVID-19 and the adoption of preventive measures. A total of 964 volunteers participated in the study. Possible predictors of risk perception were identified through a hierarchical multiple linear regression analysis, including sociodemographic, epidemiological and, most of all, psychological factors. A path analysis was adopted to probe the possible mediating role of risk perception on the relationship between the independent variables considered and the adoption of preventive measures.

**Results:** Focusing on the psychological predictors of risk perception, high levels of anxiety, an anxious attachment, and an external locus of control predicted higher perceived risk. Conversely, high levels of openness personality and of avoidant attachment predicted a lower perception of risk. In turn, the higher was the perceived risk the higher was the adoption of precautionary measures. Furthermore, psychological factors influenced the adoption of preventive behaviors both directly and indirectly through their effect on risk perception.

**Conclusions:** Our findings might be taken into high consideration by stakeholders, who are responsible for promoting a truthful perception of risk and proper compliance with precautionary measures.

## Introduction

Individually performed preventive measures are crucial for the containment of COVID-19; however, people engage in these behaviors to a dissimilar extent. This might be related to a different *perception of risk* (Brug et al., 2009), a complex phenomenon that includes both the perceived likelihood of getting sick (*personal vulnerability*) and the perceived harmfulness for one's health (*disease severity*) (Rogers, 1975; Sheeran and Abraham, 1996). The perceived risk has been positively associated with people's adherence to precautionary measures during previous respiratory infectious outbreaks (Bish and Michie, 2010) and also COVID-19 pandemic (Niepel et al., 2020; Wise et al., 2020; Yildirim et al., 2021). Conversely, individuals who perceive a low risk might not sufficiently engage in preventive behaviors, jeopardizing their own and others' health; for instance, unrealistic optimism about the likelihood of getting sick with COVID-19 in comparison to peers has been reported (Dolinski et al., 2020; Monzani et al., 2021). Yet, also a disproportionate perception of risk might be unsafe, leading to intense psychological distress (Blakey and Abramowitz, 2017) and favoring the adoption of ineffective or unnecessary preventive behaviors (Wang et al., 2020).

The keystone is a perception of risk that matches the *real* threat and that promotes an *optimal* engagement in preventive actions: indeed, risk perception can be modulated. Intense exposure to disease-related information through the media influenced the perception of risk for other respiratory infectious diseases (Barennes et al., 2010; Han et al., 2014; Choi et al., 2017), whereas the government's health communications have been effective in raising awareness about the risk for COVID-19 (Wise et al., 2020). The understanding of the factors that shape risk perception is, thus, fundamental because it might help to identify those targets more in need of a risk re-appraisal and requiring an extra communicative effort.

Most of the previous evidence about risk perception for respiratory infectious diseases focused on *sociodemographic* and *epidemiological* factors; female gender, older age, poor health, and lower education have been related to higher perceived risk for COVID-19 (Casanova et al., 2020; Costa, 2020; Dolinski et al., 2020; González-Olmo et al., 2020; He et al., 2021). Living in most affected areas (Ibuka et al., 2010; Alqahtani et al., 2017) or with people with chronic diseases (He et al., 2021), knowing someone affected (Kim et al., 2015), professional exposure to the disease (Peres et al., 2020; He et al., 2021; Karasneh et al., 2021) and trust in stakeholders (Choi et al., 2017; Wang et al., 2018; Jang et al., 2020) increase the perceived threat for COVID-like diseases.

Only a minority of studies considered the *psychological* factors possibly affecting risk perception; those who were confident that they can cope with the disease reported a lower risk perception (Han et al., 2014; Choi et al., 2017; Commodari, 2017) whereas overall psychological distress was positively related to risk perception for respiratory infectious diseases (Barr et al., 2008). Also, anxiety—but not depressive symptoms—was positively associated with risk perception for COVID-19 (Pérez-Fuentes et al., 2020). Personality traits have been related to risk perception concerning several possible hazards, including those related to individuals' physical health (Sjöberg and Wåhlberg, 2002; Sjöberg, 2003; Chauvin et al., 2007). In Italy, risk perception for influenza pandemic was reported to be higher in those individuals who scored lower in “dynamicity,” and “imagination” and higher in “vulnerability” (i.e., feeling sad, guilty, worried) and “conscientiousness” (Commodari, 2017); also, empathy and imagination positively predicted the perceived risk for infectious diseases in general (Commodari et al., 2020). Concerning COVID-19, people who scored higher on agreeableness perceived lower risk (Rammstedt et al., 2021); conversely, higher emotionality predicted higher risk perception (Oljača et al., 2020). Higher neuroticism was associated with higher concerns (Aschwanden et al., 2021).

Furthermore, it is conceivable that the psychological dimensions related to how people face threatening and stressful situations might have a role in the perception of risk for COVID-19. The process of coping refers to the selection and execution of certain responses to overcome demanding circumstances (Lazarus, 1966). Dealing actively with the stressor and related emotions, that is an approach, active, coping, is generally considered more adaptive and effective than eluding the situation, i.e. an avoidant coping (Carver et al., 1989). Risk perception has been positively associated with the adoption of active coping strategies (Li et al., 2021), including emotion-focused, problem-focused and meaning-focused strategies (Krok and Zarzycka, 2020). Adaptive coping mediated and swapped the negative relationship between high risk perception for COVID-19 and low people's psychological well-being (Krok and Zarzycka, 2020); in turn, risk perception mediated the association between lower social support and higher adoption of active strategies to cope with COVID-19 (Li et al., 2021). Nevertheless, it is not clear how the adoption of less adaptive strategies (i.e., avoidant coping) may interplay with risk perception.

Also, fearful and stressful situations activate cognitive-affective schemas related to people's attachment (Bowlby, 1979). A secure attachment favors the development of self-worth and self-competence (Mikulincer and Shaver, 2019) and promotes the adoption of more adaptive problem-focused strategies (Simpson and Rholes, 2018). Therefore, attachment might modulate people's capability to cope with infectious outbreaks, shaping the perceived risk by affecting the perceived vulnerability and self-efficacy. Nevertheless, the possible relationship between risk perception and attachment dimensions has been relatively neglected in the literature.

Finally, the health-related locus of control might influence risk perception because it affects the perceived control over one's health, i.e., whether itis determined by internal or external causes. For instance, individuals who believe that their health mainly depends on their own choices, that is an internal locus of control, showed a more accurate perception of risk for HIV (Crisp and Barber, 1995) whereas those who think that their health is determined by external forces (i.e., an external locus of control) perceived higher vulnerability (Heaven et al., 1992). This might be true also for other infectious diseases, including COVID-19.

The aim of this investigation is two-fold. Primarily, extending the previous evidence about the possible predictors of risk perception for COVID-19 including both sociodemographic and epidemiological variables but focusing on several psychological dimensions. According to the literature background previously illustrated, we hypothesize that lower perceived risk might be related to higher *self-efficacy* and higher levels of *extraversion, openness, emotional stability*, and *agreeableness* dimensions of personality. Also, we hypothesize that individuals who adopt *avoidant coping* strategies and have an insecure—*avoidant attachment* might elude the stressor, perceiving lower risk. Conversely, we expect that more *anxious* and *conscious* individuals and those who have an insecure—*anxious attachment* and an *external locus of control* might perceive higher risk; likewise, we hypothesize that people relying on *active coping strategies* might be more focused and fully aware of the potential threat, perceiving higher risk.

Secondarily, we aim at investigating the relationship between the possible predictors of risk perception for COVID-19 and the adoption of preventive measures, considering the possible mediating role of risk perception. Indeed, we expect that higher risk perception will be associated to a higher adoption of preventive measures and that the demographic, epidemiological and psychological dimensions influencing the perceived risk will indirectly also affect people's behaviors.

## Method

### Participants

A total of 964 participants volunteered for this study. After removing few duplicated cases, 911 Italian participants were included in the analyses (mean age: 41.61 ± 13.73, age range: 18–82; 699 females, 76.7%; see Table 1). Most of the sample was living in Northern Italy (*n* = 794; 87.2%), attended at least high school (*n* = 305; 33.5%) or had a University or higher degree (*n* = 571; 62.7%), was in a relationship or married (*n* = 666; 73.1%). Inclusion criteria were being aged 18 or more and being Italian native speakers. The study was conducted in accordance with the ethical principles of the Declaration of Helsinki and was approved by the Ethical Committee of the IRCCS, Istituto Auxologico Italiano (Milan, Italy).

**Table 1:** Means, standard deviations, frequencies, percentages for all sociodemographic, epidemiological, and psychological variables, and their associations with Risk Perception (*N* = 911).

**Variable(s)**	**Frequency (%) or mean (SD*)***	***r* with Risk perception**
Risk Perception, *mean (SD)*	76.14 (30.13)	/
Age, *mean (SD)*	41.61 (13.73)	0.015
Sex—Men	699 (76.7%)	−0.080^*^
Filling out the battery during Lockdown	595 (65.3%)	0.003
Living in area with more than 1% of infected Population	316 (34.7%)	0.030
Education (University)	571 (62.7%)	0.025
Civil Status (Married/in a Relationship)	666 (73.1%)	−0.009
Living with people at High Risk	205 (22.8%)	0.099^**^
Physical Health, *mean (SD)*	4.00 (0.72)	−0.120^**^
Chronic Diseases	203 (22.3%)	0.083^*^
COVID-like symptoms	165 (18.1%)	0.114^**^
COVID-19 diagnosis	9 (1.0%)	0.022
Cases among close friends or relatives	382 (41.9%)	0.066^*^
Deaths among close friends or relatives	198 (21.7%)	0.126^**^
Working near/in contact with COVID-19 patients	104 (11.4%)	0.230^**^
Adequacy of received info, *mean (SD)*	10.05 (2.80)	0.063
Trust in Institutions, *mean (SD)*	3.30 (1.17)	0.047
Adoption of Preventive Measures, *mean (SD)*	4.48 (0.70)	0.178^**^
ECR-12 Anxiety, *mean (SD)*	3.22 (1.46)	0.176^**^
ECR-12 Avoidance, *mean (SD)*	2.51 (1.40)	−0.039
PHQ-9, *mean (SD)*	5.88 (4.40)	0.186^**^
GAD-7, *mean (SD)*	5.62 (4.23)	0.219^**^
GSE, *mean (SD)*	37.67 (6.82)	−0.060
TIPI Extroversion, *mean (SD)*	3.95 (1.49)	−0.019
TIPI Agreeableness, *mean (SD)*	5.22 (1.09)	0.039
TIPI Conscientiousness, *mean (SD)*	5.39 (1.20)	0.005
TIPI Emotional Stability, *mean (SD)*	4.46 (1.38)	−0.087^**^
TIPI Openness, *mean (SD)*	4.48 (1.16)	−0.080^*^
Brief-COPE Approach, *mean (SD)*	21.98 (4.61)	0.055
Brief-COPE Avoidant, *mean (SD)*	34.07 (6.12)	0.163^**^
H-LoC Internal, *mean (SD)*	32.3 (4.81)	0.012
H-LoC External God, *mean (SD)*	4.35 (2.44)	0.103^**^
H-LoC External Others, *mean (SD)*	5.35 (2.37)	0.138^**^

* * = correlation is significant at the.05 level*;

** * = correlation is significant at the.01 level. For the variable Sex, the reference category was “Men”. ECR-12, experiences in close relationships–12; PHQ-9, patient health questionnaire–9; GAD-7, general anxiety disorder scale–7; GSE, general self-efficacy scale; TIPI, ten-item personality inventory; Brief-COPE, brief—coping orientation to problems experienced; H-LoC, health-related locus of control scale*.

### Procedure

An on-line, cross-sectional survey was conducted between May and July 2020, which corresponded approximately with the end of the first lockdown in Italy and the progressive flattening of the epidemic curve. A snowball convenience sampling was adopted. The survey was distributed through institutional media, social networks, and authors' personal and professional contacts. Before filling up the questionnaire, participants gave their digital informed consent, declaring to be of legal age and to have read and to accept the privacy regulation. The battery of questionnaires was created using Google Forms (©Google). The participation was anonymous.

### Measures

Sociodemographic and Epidemiological Information. Age, sex, education, place of living, employment status, family status, self-reported health status, presence of COVID-19 symptoms or diagnosis, exposure to people affected by COVID-19, degree of adherence and motivation to adopt preventive behaviors, and perceived adequacy of disease-related information were investigated through an *ad-hoc* questionnaire.

Risk Perception. A questionnaire used in a previous study on risk perception for avian influenza (Cui et al., 2017) was adapted for COVID-19. The original items were translated into Italian using a forward and backward translation procedure (Beaton et al., 2000). The questionnaire includes 8 items, rated on a 5-point Likert-type scale (1 = totally disagree; 5 = totally agree). Two items assess the perceived likelihood of getting sick, that is the personal *Vulnerability* and three items investigate the perceived harmfulness of COVID-19 for one's health, which is the disease *Severity*. The questionnaire was developed *ad-hoc* for the present study; an English version of this measure is reported in Supplementary Table 1.

Depressive Symptoms—Patients Health Questionnaire 9 (PHQ-9) (Mazzotti et al., 2003). This is a 9-item self-report measure of depressive symptomatology in the last two weeks. In the current study, the Cronbach's Alpha was good (α = 0.84).

Anxious Symptoms—General Anxiety Disorder Scale 7 (GAD-7) (Spitzer et al., 2006). This is a seven-item self-report measure of anxious symptoms in the last two weeks. In the current study, the Cronbach's Alpha was good (α = 0.89).

Perceived Self-Efficacy—General Self-efficacy Scale **(GSE)** (Sibilia et al., 1995). This is a 10-item self-report measure assessing a person's sense of personal competence to effectively manage stressful situations. In the current study, the Cronbach's Alpha was excellent (α = 0.90).

Attachment Dimensions—Experiences in Close Relationships 12 (ECR-12) (Brugnera et al., 2019). This is a 12-item self-report measure of two dimensions of attachment to romantic partners, namely attachment *Avoidance* (six items) and attachment *Anxiety* (sixitems). In this study, the Cronbach's alphas of the two subscales were good to excellent (α = 0.91 for attachment avoidance and α = 0.85 for attachment anxiety).

Personality Traits—Ten-Item Personality Inventory (TIPI) (Chiorri et al., 2015). This is a 10-item measure of five personality traits (namely *Extroversion, Agreeableness, Conscientiousness, Emotional Stability*, and *Openness* to experiences) according to the Big Five personality dimensions. In this study, the inter-item correlation coefficients were good (range: 0.18–0.44). Inter-item correlation coefficients in the range of 0.15–0.50 indicate good internal consistency of a scale (Clark and Watson, 1995).

Health-Related Coping Styles—Brief Coping Orientation to Problems Experienced (Brief-COPE) (Monzani et al., 2015). This is a 28-item measure designed to measure *Avoidan*t and *Approach* (i.e., active) coping styles to health-related stressful life events. In this study, the Cronbach's alphas of the two subscales were fair to good (α = 0.64 for Avoidant and α = 0.80 for Approach).

Locus of Control—Health Locus of Control Scale (H-LoC) (Donizzetti and Petrillo, 2015). This is a 13-item self-report measure of the participants' perception to have direct or indirect control over their health, namely an internal or external locus of control. The H-LoC is composed of three subscales: *Internal LoC* (eight items), *External LoC God* (i.e., control is attributed to transcendental entities; two items), *External LoC Others* (control is attributed to others significant people; three items). In this study, the Cronbach's alphas of the three subscales were acceptable to good (α range: 0.71–0.87).

### Statistical Analysis

An a-priori power analysis showed that, given an α value of 0.05 and a power (β) of 0.80, a sample size of 954 would have allowed detecting a small effect size (*f*^2^ = 0.02) for regression analysis with 15 predictors (i.e., the psychological IVs entered in the model; see below). Thus, our study was adequately powered. Data collected were initially analyzed using descriptive and univariate statistics, including means, standard deviations, frequencies, percentages, and Pearson's *r* correlation coefficients. As a preliminary analysis, the internal validity of the Risk Perception scale was evaluated through an Exploratory Factorial Analysis (EFA), whose details are provided in Supplementary Material. The EFA identified two components: *Severity* (three items) and *Vulnerability* (two items; see Supplementary Tables 2, 3 for all results). Both scales had good internal reliability (α of 0.89 for Severity; inter-item correlation of 0.38 for Vulnerability). In accordance with previous literature (De Zwart et al., 2007, 2009), the product of the *Severity* and *Vulnerability* was computed, obtaining a new scale called “*Risk Perception*,” which was used in all analyses.

To test our first hypothesis, a hierarchical multiple linear regression analysis was run to identify the significant predictors of Risk Perception. Our dependent variable (DV) was risk perception, while the independent variables (IVs) at block 1 were age, sex, having filled out the questionnaires before or after the lockdown, percentage of infected population in the living area above or below 1%, education (dichotomized as above or below high school), family status (dichotomized as single/divorced/widowed, or married/engaged), living with people at high risk, the perceived quality of physical status, having a chronic physical condition, having experienced COVID-like symptoms, swab outcome for a diagnosis of COVID-19, having had relatives/ close friends with a diagnosis of COVID-19, having experienced a COVID-related death among relatives/close friends, working in contact with COVID-19 patients, perceived adequacy of disease-related information, and the general trust in institutions for containing COVID-19 spread. Further, in Block 2 all the above-mentioned psychological dimensions (anxious and depressive symptoms, perceived self-efficacy, attachment dimensions, personality traits, health-related coping styles, and locus of control) were entered. This analytical approach allowed us to examine the predictive role of psychological dimensions over and above all other sociodemographic and epidemiological variables. As a measure of effect size, the partial correlation coefficient *r* for each IVs, and the adjusted percentage of explained variance (R^2^) for each block is reported; effect sizes were interpreted according to guidelines (Cohen, 1988). As regards the assumptions of multivariate analyses, no univariate outliers were identified. Several variables were transformed through square-root, log10, or reflect and inverse transformations to correct their non-normal distributions. The presence of multivariate outliers was evaluated and a total of four cases were identified, which were removed from both the regression and the path analysis (Tabachnick et al., 2007). Further, the assumption of multicollinearity was assessed by computing and examining both the Variance Inflation Factor (VIF) and Tolerance values. The presence of strong multicollinearity is suggested by values above 10 and below 0.1, respectively (Lin, 2008).

Finally, the mediating role of Risk Perception on the relationship between sociodemographic, epidemiological, and psychological predictors (the IVs) and the adoption of preventive measures (the DVs)—our secondary hypothesis—was tested through a path analysis with observed variables. Only those predictors that were significant in the regression analysis were entered as IVs. Parameter estimates were computed using a maximum likelihood estimation method, while an optimal model fit was evaluated using the following criteria: an RMSEA of 0.05 or less, an upper RMSEA's 90% CIs of 0.08 or less, a CFI, and a TLI of 0.95 or more, and a SRMR of 0.05 or less. The magnitude of path coefficients was interpreted according to Cohen's criteria (Cohen, 1988). Indirect (i.e., mediated) effects and their standard errors were further computed using a bootstrap procedure, saving parameter estimates drawn from 10,000 bootstrap samples. If the 95% confidence intervals (CI) of these estimates do not include zero, then the indirect (mediated) effect is statistically significant at the 0.05 level (Byrne, 2013). Analyses were performed using SPSS and AMOS version 26.0.

## Results

Descriptives of all sociodemographic, epidemiological and psychological variables and their zero-order correlations with risk perception are reported in Table 1. Correlations evidenced that those living with people at high risk, those who experienced COVID-like symptoms, those who had cases/deaths among friends or relatives, those who worked near/in contact with COVID-19 patients, individuals who adopted more often the preventive measures, those with higher levels of attachment anxiety, anxious and depressive symptoms, with an avoidant coping style and with an external health-related locus of control, reported higher levels of risk perception. On the contrary, men, those with a better self-reported physical health, with higher emotional stability and openness personality traits reported lower levels of risk perception. All effect sizes were trivial to small.

A table with zero-order correlations among all variables used in this study is provided in Supplementary Material (see Supplementary Table 4).

### Predictors of Risk Perception

The significant predictors of Risk Perception were identified through a hierarchical multiple linear regression analysis. Results evidenced that at Block 1, sociodemographic and epidemiological variables contributed significantly to the regression model, *F*_(16,847)_ = 6.537; *p* < 0.001 and accounted for 9.3% of the variation in the dependent variable. Introducing the psychological predictors (e.g., attachment insecurity, anxious and depressive symptoms) explained an additional 7.7% of variation in risk perception and this change in R^2^ was significant, *F*_change(15,832)_ = 6.248; *p* < 0.001. In both Blocks, the assumption of multicollinearity was met (Block 1: Tolerance = 0.718–0.977; VIF = 1.024–1.393; Block 2: Tolerance = 0.338–0.950; VIF = 1.053–2.955).

Examining the significant predictors at Block 2, those who experienced COVID-related deaths among close friends or relatives, who were working near/in contact with COVID-19 patients, who have received adequate information about COVID-19, those with higher levels of attachment anxiety, anxious symptoms, and of a health-related external locus of control (others), experienced a higher risk perception, while controlling for all other variables in the model. On the contrary, those with higher levels of openness and attachment avoidance experienced lower levels of risk perception, over and above all other variables in the model. All effect sizes (partial *r*) were trivial to small (see Table 2 for all regression values).

**Table 2:** Unstandardized B, Standard Errors, Standardized Beta, *t*-values, *p*-values and partial r correlation coefficient of the sociodemographic, epidemiological and psychological predictors of Risk Perception, in the total sample of 907 Italian participants.

**Block**	**Predictors**	**B**	**SE**	**Beta**	***t*-value**	***p*-value**	**Partial *r***
Block 1	Total Adjusted R^2^ =0.093						
	(Constant)	8.891	0.507		17.553	<0.001	
	Filling out the battery during Lockdown	0.013	0.128	0.004	0.102	0.919	0.004
	Age	−0.002	0.005	−0.017	−0.477	0.633	−0.016
	Sex—Men	−0.173	0.137	−0.042	−1.269	0.205	−0.044
	Living in area with more than 1% of infected Population	−0.101	0.142	−0.027	−0.712	0.477	−0.024
	Education (University)	0.115	0.123	0.032	0.936	0.350	0.032
	Civil Status (Married/in a Relationship)	−0.082	0.130	−0.021	−0.629	0.529	−0.022
	Living with people at high risk	0.376	0.137	0.090	2.746	0.006	0.094
	Physical Health	−0.296	0.087	−0.121	−3.398	0.001	−0.116
	Chronic Diseases	0.179	0.154	0.042	1.161	0.246	0.040
	COVID-like symptoms	0.318	0.153	0.071	2.077	0.038	0.071
	COVID-19 diagnosis	−0.129	0.605	−0.007	−0.213	0.832	−0.007
	Cases among close friends or relatives	−0.106	0.132	−0.030	−0.803	0.422	−0.028
	Deaths among close friends or relatives	0.533	0.157	0.126	3.403	0.001	0.116
	Working near/in contact with COVID-19 patients	1.213	0.184	0.223	6.591	<0.001	0.221
	Adequacy of received info	0.043	0.023	0.069	1.891	0.059	0.065
	Trust in institutions	0.053	0.055	0.035	0.957	0.339	0.033
Block 2	Total Adjusted R^2^ =0.17; Adjusted R^2^ change =0.077						
	(Constant)	6.277	1.289		4.871	<0.001	
	Filling out the battery during lockdown	0.068	0.124	0.019	0.552	0.581	0.019
	Age	0.009	0.005	0.072	1.885	0.060	0.065
	Sex—Men	0.008	0.140	0.002	0.054	0.957	0.002
	Living in area with more than 1% of infected population	0.010	0.137	0.003	0.073	0.942	0.003
	Education (University)	0.213	0.120	0.059	1.766	0.078	0.061
	Civil status (married/in a relationship)	−0.084	0.133	−0.021	−0.631	0.529	−0.022
	Living with people at high risk	0.251	0.133	0.060	1.894	0.059	0.066
	Physical health	−0.168	0.089	−0.068	−1.887	0.060	−0.065
	Chronic diseases	0.276	0.149	0.065	1.858	0.064	0.064
	COVID-like symptoms	0.220	0.149	0.049	1.481	0.139	0.051
	COVID-19 diagnosis	−0.105	0.585	−0.006	−0.179	0.858	−0.006
	Cases among close friends or relatives	−0.159	0.128	−0.045	−1.245	0.214	−0.043
	Deaths among close friends or relatives	0.476	0.152	0.113	3.133	0.002	0.108
	Working near/in contact with COVID-19 patients	1.317	0.178	0.242	7.398	<0.001	0.248
	Adequacy of received info	0.046	0.022	0.073	2.072	0.039	0.072
	Trust in institutions	0.044	0.054	0.030	0.829	0.408	0.029
	ECR-12 anxiety	0.504	0.145	0.121	3.467	0.001	0.119
	ECR-12 avoidance	−0.560	0.266	−0.075	−2.104	0.036	−0.073
	PHQ-9	0.044	0.112	0.021	0.392	0.695	0.014
	GAD-7	0.319	0.114	0.149	2.793	0.005	0.096
	GSE	−0.003	0.010	−0.011	−0.265	0.791	−0.009
	TIPI extroversion	0.024	0.040	0.021	0.601	0.548	0.021
	TIPI agreeableness	0.065	0.055	0.040	1.174	0.241	0.041
	TIPI conscientiousness	0.163	0.161	0.035	1.017	0.309	0.035
	TIPI emotional stability	0.027	0.053	0.021	0.497	0.619	0.017
	TIPI openness	−0.171	0.052	−0.115	−3.268	0.001	−0.113
	Brief-COPE approach	−0.037	0.083	−0.017	−0.446	0.656	−0.015
	Brief-COPE avoidant	0.248	0.138	0.070	1.799	0.072	0.062
	H-LoC Internal	0.124	0.065	0.063	1.890	0.059	0.065
	H-LoC external god	0.304	0.369	0.028	0.822	0.411	0.029
	H-LoC external others	1.533	0.712	0.074	2.153	0.032	0.074

*ECR-12, experiences in close relationships−12; PHQ-9, patient health questionnaire−9; GAD-7, general anxiety disorder scale−7; GSE, general self-efficacy scale; TIPI, ten-item personality inventory; Brief-COPE, brief—coping orientation to problems experienced; H-LoC, health-related locus of control scale*.

### Mediation Model

The mediating role of risk perception on the association between sociodemographic, epidemiological, and psychological predictors and the adoption of preventive measures was tested through a path analysis. Firstly, the mediation model had a good fit to the data: χ^2^ (15) = 17.914, *p* =0.267; *RMSEA* =0.015, 90% CIs (< 0.01, 0.04); *CFI* = 0.99; *TLI* =0.98; *SRMR* = 0.020. As evidenced in Supplementary Table 5, having experienced deaths among close friends or relatives, working near/in contact with COVID-19 patients, higher levels of external (others) health-related locus of control, of anxious symptoms, and attachment anxiety had a significant positive direct effect on risk perception. Further, openness had a significant negative direct effect on risk perception. As regards the direct paths on the variable “adoption of preventive measures,” risk perception and adequacy of information had significant and positive direct effects on the dependent variable, while external (others) health-related locus of control and attachment avoidance had significant, negative direct effects on it. All other direct paths were non-significant. Effect sizes (Beta) were trivial to small.

**Figure 1:**
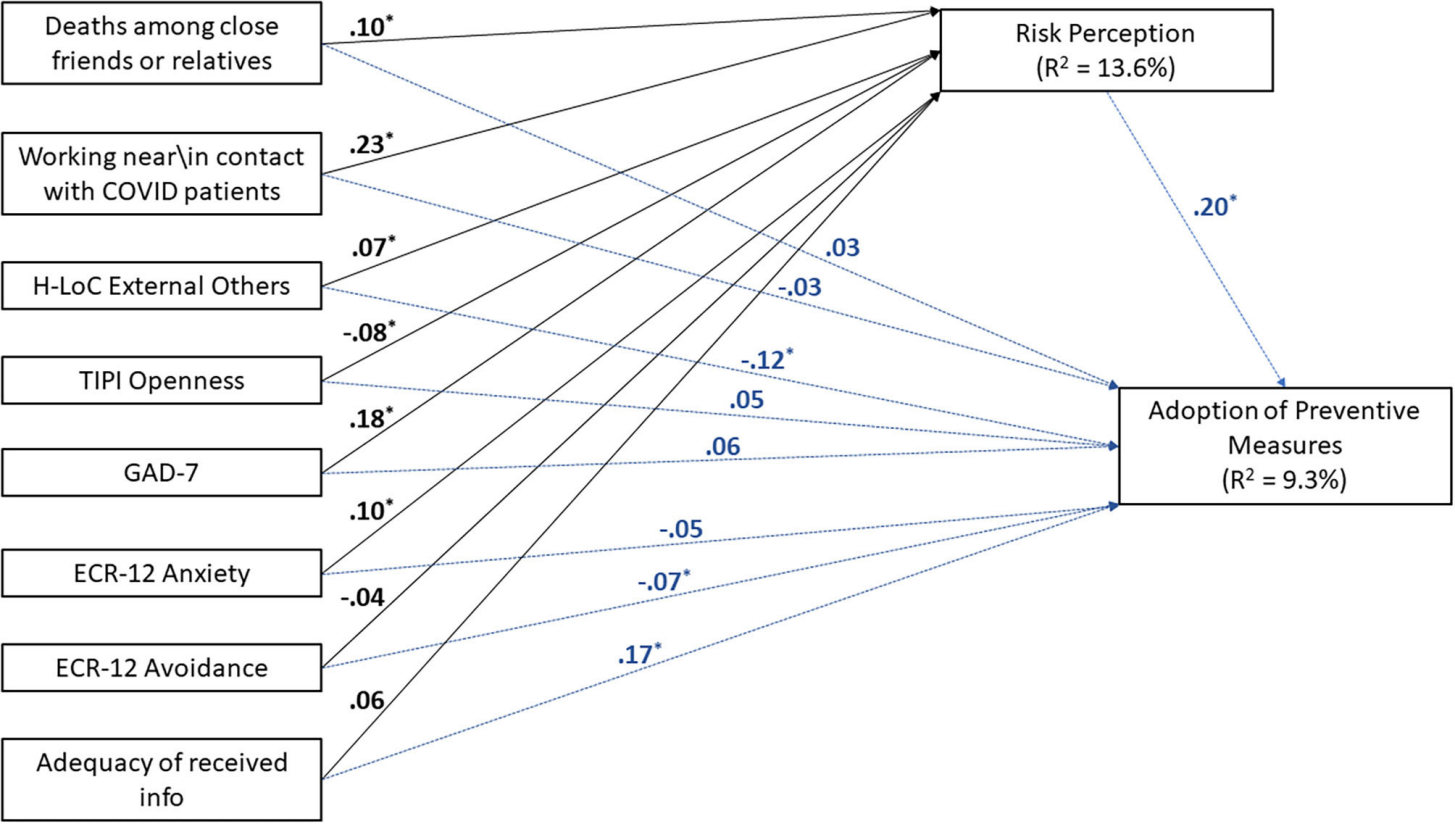
Standardized direct paths of the mediation model (N = 907). Dashed blue lines (and blue parameters) represent the *direct* paths of all IVs on the dependent variable “Adoption of Preventive Measures.” Regarding indirect effects, the variables (others) health-related locus of control, anxious symptoms, attachment anxiety, working near/in contact with COVID-19 patients, and having experienced one or more deaths among close friends or relatives (due to COVID) had a significant, positive indirect effect on the adoption of preventive measures through risk perception, while openness had a significant indirect negative effect on the dependent variable (see Supplementary Table 6 for more information). H-LoC = Health-related Locus of Control scale; TIPI = Ten-Item Personality Inventory; GAD-7 = General Anxiety Disorder Scale–7; ECR-12 = Experiences in Close Relationships−12.

Bias-corrected bootstrapped tests of mediation evidenced that (others) health-related locus of control, anxious symptoms, attachment anxiety, working near/in contact with COVID-19 patients, and having experienced one or more deaths among close friends or relatives (due to COVID) had a significant, positive indirect effect on the adoption of preventive measures through risk perception, while openness had a significant indirect negative effect on the dependent variable (see Figure 1; Supplementary Table 6 for all effects, 95% CIs and *p*-values). All effect sizes were small. That is, attachment anxiety -for example- had an indirect effect on the adoption of preventive measures through a sequence of casual steps in which attachment anxiety increased risk perception, which in turn increased the adoption of preventive measures. The independent variables accounted for ~13.6% of the variance in risk perception, while the entire model accounted for 9.3% of the variance in the adoption of preventive measures.

## Discussion

Risk perception for COVID-19 was investigated in a sample of 911 Italian adults, within the last weeks of the first national lockdown and the progressive flattening of the epidemic curve. Our first aim was to probe which sociodemographic, epidemiological, and especially psychological factors significantly predict risk perception.

Considering the possible sociodemographic and epidemiological predictors (weighted for the effect of psychological factors, i.e. in the Block 2 of regression analysis), the experience of deaths among relatives or close friends, working in contact with COVID-19 patients, and the perceived adequacy of the information received significantly predicted a higher risk perception. Experiencing the loss of significant others conceivably increases the perceived proximity of the threat, consequently, it possibly amplifies the perception of its dangerousness. Similarly, being in contact with people affected by COVID-19 likely increases the perceived exposure to the threat and, thus, the possibility of being infected; moreover, dealing with affected people gives a direct experience of COVID-19 potential harmfulness. These results are in line with the previous evidence (Kim et al., 2015; Alqahtani et al., 2017; Peres et al., 2020; He et al., 2021; Karasneh et al., 2021); conversely, experiencing COVID-19 symptoms and being diagnosed with COVID-19 did not influence risk perception. Nevertheless, very few participants reported COVID-19 symptoms and even fewer received a formal diagnosis, likely affecting the possibility to detect a significant relationship between these variables and risk perception.

Focusing on the perceived adequacy of the information received about COVID-19 symptoms, prognosis, and how to prevent the contagion, the more people believed to be well-informed, the more they perceived higher risk. Feeling confident about one's knowledge possibly encouraged people to “take it seriously,” perceiving higher risk. Similarly, relying on official sources of communication and being frequently exposed to disease-related information through the media have been related to higher risk perception for COVID-19 (Huynh, 2020; He et al., 2021; Karasneh et al., 2021). This is especially relevant to be acknowledged by stakeholders, who should try to promote the clearest and most coherent risk-communications.

According to the regression analysis (Block 2), none of the other sociodemographic and epidemiological variables influenced risk perception for COVID-19. This might be surprising since age (Bruine de Bruin, 2020; González-Olmo et al., 2020; He et al., 2021), gender (Dolinski et al., 2020; González-Olmo et al., 2020; Taghrir et al., 2020; He et al., 2021), education (Costa, 2020), living with vulnerable people (He et al., 2021), and the perceived health status (Casanova et al., 2020; Costa, 2020; González-Olmo et al., 2020) have been related to risk perception for COVID-19 in other studies. Nevertheless, preliminary correlational analyses showed that risk perception *does* increase among women, in those people who reported poor health or had chronic diseases, and among those living with vulnerable people or who experienced COVID-like symptoms; however, the effect of these variables was no more significant when considering simultaneously multiple possible predictors of risk perception, including the psychological factors. This suggests that variables other than sociodemographic/epidemiological factors may better explain part of the variance in risk perception. In this regard, only a minority of the studies showing a relationship between risk perception for COVID-19 and sociodemographic factors adopted a regression analysis approach (Bruine de Bruin, 2020; He et al., 2021), and none of them considered the possible interplay of psychological factors, except for anxiety and depressive symptoms (Bruine de Bruin, 2020).

Filling up the questionnaire during the lockdown and living in the most affected areas did not influence risk perception, suggesting that the risk appraisal was relatively stable in time and space. This result contrasts with previous findings on other respiratory infectious diseases (De Zwart et al., 2009; Ibuka et al., 2010; Alqahtani et al., 2017); however, the intense and persistent media coverage possibly contributed to level out people's perception across different regions and times. Finally, people's trust in institutions for managing the contagion was low on average, but not related to risk perception. Discordant results have been reported in the literature on this topic (Choi et al., 2017; Yang and Cho, 2017; Jang et al., 2020), thus, future studies might probe further this issue.

Considering the psychological predictors of risk perception, which was our main interest, no association between general self-efficacy and the perceived risk was reported. This result contrasts with our hypothesis and with the previous evidence of a negative association between general self-efficacy and risk perception for influenza pandemic in Italy (Commodari, 2017). However, our finding is in line with Kim and Kim's (2018), who reported no association between disease-related self-efficacy and risk perception for MERS. Furthermore, the COVID-19 pandemic is far more dramatic than previous infectious outbreaks; people may feel especially powerless despite their perceived personal resources, thus, the possible effect of perceived self-efficacy on risk perception might be dampened in a similar scenario.

As expected, high levels of anxiety predicted a higher perceived risk, but depressive symptoms did not. This observation matches with other recent findings on COVID-19 (Pérez-Fuentes et al., 2020), suggesting that anxiety and depression might have a dissimilar effect on the perceived threat. Anxious people might be more predisposed to overreact in the face of a pandemic since even in non-threatening situations they show excessive apprehension, worries, and they experience unjustified fear (American Psychiatric Association, 2013). Conversely, depressive symptoms include apathy, loss of interest in the self and others, and feeling of worthlessness (American Psychiatric Association, 2013); thus, a reduced focus on and awareness of the external world might soften the perceived relevance of the threat. Previous studies adopted a non-specific measure of psychological distress, merging anxious and depressive symptoms (Barr et al., 2008; Jacobs et al., 2010); thus, a dissimilar prevalence of either anxiety or depression in those samples might have influenced the results.

Our hypothesis concerning risk perception and personality dimensions was only partially supported. Our results showed that greater levels of openness predicted a reduced perception of risk, meaning that high levels of intellect, reflection, creativity, and imagination lowered the perceived risk. Greater creativity might favor figuring out several “way outs” and, possibly, more alternative optimistic future scenarios, thus reducing the perceived risk. This is in line with the record that more “imaginative” people perceived a lower risk for COVID-like diseases (Commodari, 2017; Commodari et al., 2020) but, contrary to expectations, none of the other personality dimensions was associate with risk perception. Heterogeneity in the assesment measures and the theoretical frameworks adopted in previous studies might explain incongruent findings. For instance, Rammstedt et al. (2021) found that risk perception was not uniformly related to all the facets of agreeableness since it was correlated mainly with the trust facet. However, the questionnaire we used did not allow such refined profiling of personality facets. Yet, research on the role of personality traits in perceiving the risk for COVID-like diseases is limited (Commodari, 2017; Commodari et al., 2020; Oljača et al., 2020; Rammstedt et al., 2021).

In contrast to our initial hypothesis, the adoption of—more effective—approach coping strategies rather than of the—less adaptive—avoidant ones did not have any effect on the perceived risk. However, participants must say how they *usually* react to health problems, but the present situation is extraordinary. Therefore, the strategies adopted to cope with the COVID-19 pandemic may differ from individuals' typical behaviors, at least partially; for instance, typical coping strategies might not be strictly related to risk perception for COVID-19 because people have been specifically instructed by authorities about how to face the outbreak.

On the other hand, as expected, an avoidant attachment was a significant, negative, predictor of risk perception, while anxious attachment was a positive predictor. In response to inconsistent and/or unresponsive caregivers during childhood, avoidant individuals have learnt how to be self-reliant, facing stressful situations by engaging in deactivating coping strategies aimed at denying the problem and suppressing negative thoughts and emotions (Pascuzzo et al., 2013). Consequently, they might deny the threat, feeling lower risk. Conversely, an anxious attachment is characterized by the adoption of emotion-focused or hyper-activating coping strategies. This behavior maintains the caregivers close but it sustains and even increases people's worries (Pascuzzo et al., 2013), who likely overreact. Furthermore, the functional role of attachment in driving people's behaviors may extend beyond the single individual. That is, individuals' attachment dimensions could influence the group's behavioral response, especially in the face of a potential threat (Ein-Dor et al., 2010, 2011). People with a high level of secure attachment have internalized an overall feeling of safety, they are self-confident, optimistic and they know how to engage in efficacious problem-solving. According to the Social Defense Theory—SDT (Ein-Dor et al., 2010), they keep calm, reassure and successfully coordinate the other members in demanding situations. However, this might not be enough when facing sudden and ambiguous threats. The SDT suggests that, when people are at risk, individuals with a high level of anxious and avoidant attachment might have a crucial—beneficial—role. Hypervigilance related to anxious attachment might favor the early detection of a potential threat; on the other hand, avoidant individuals who usually rely on quick, cold, fight-or-flight responses, might be more prone to identify efficacious solutions to protect themself, but possibly also the others (Ein-Dor et al., 2010). Therefore, the association observed between the level of anxious and avoidant attachment and risk perception might go beyond the single individual, influencing how closer people perceived the risk. This might be especially relevant when considering collective and pervasive threats that require a cooperative response, such as COVID-19.

Finally, and in line with our expectations, the more people believe that their health depends on inscrutable forces, such as fate and God, or on other people (i.e., they have an external locus of control) the more they perceive higher risk. In other words, they believe that their health is unrelated to their own choices, likely feeling no control over the contagion and, thus, perceiving higher risk.

Once the possible predictors of risk perception were detected, our second aim was to probe whether these factors also influence the adoption of preventive behaviors and, if so, whether their effect is mediated by the perceived risk. The mediation model showed that perceived risk and the (perceived) adequacy of the disease-related information received directly favored the adoption of preventive measures, whereas external (others) health-related locus of control and attachment avoidance directly reduced people's compliance with protective behaviors. In other words, it seems that being adequately informed about COVID-19 encourages people to comply with the containment measures, possibly because of a better understanding of the disease-related outcomes and of the rationale behind the actions adopted by the government. On the other hand, if people believe they cannot do anything on their own to avoid the contagion (because others determine their health), they might adopt a fatalistic approach, considering it pointless to engage in the recommended behaviors. Indeed, according to the learned helplessness theory, people exposed to uncontrollable events learn that outcomes do not depend on their responses, leading to the expectation that any response will be futile (Seligman, 1975). An external locus of control has been reported to favor this process (Cohen et al., 1976) and also to be associated with higher hopelessness, that is the tendency to have a negative and pessimistic vision of the future and lose motivation (Plahuta et al., 2002). Finally, as previously mentioned, people with an avoidant attachment tend to deny the relevance of problematic situations, therefore they may not be sufficiently motivated to protect themselves or others. Furthermore, our mediation model showed that some of those factors that do not directly influence the adoption of preventive measures indirectly affect people's compliance through their effect on the perceived risk. High levels of anxious symptoms, attachment anxiety, working in contact with COVID-19 patients, and the experience of deaths among significant others increased the perceived risk for COVID-19 that, in turn, leads to greater adoption of preventive measures. Conversely, a high level of openness reduces people's adherence to precautionary measures by lowering the perceived risk. Finally, it is worth noting that having high levels of external (others) health-related locus of control indirectly *increases* people's compliance with the preventive measure by increasing the perceived risk but, as previously mentioned, it directly discourages people's engagement in these behaviors. This and the above findings suggest that people's engagement in preventive behaviors is an intricate phenomenon, related to several, interplaying, factors. Risk perception seems to have a role; however, it is a complex domain itself. Indeed, when investigating the possible predictors of risk perception, the variables considered explained only a small percentage of the variance (i.e., 17%), thus, many other, neglected, factors possibly explain risk perception.

Concerning the possible limitations of our study, the adoption of an on-line survey may limit the coverage of the questionnaire, and especially it might have discouraged the recruitment of elders, people with low education, and of those who have no easy access to the Internet. Moreover, in our convenience sample the prevalence of certain sociodemographic features might not be balanced or representative of the Italian population. For instance, women and people with high education (i.e., a University degree or higher) were overrepresented (ISTAT, 2020) and most of the sample lived in Northern Italy, which was far more affected during the first wave of the pandemic. This approach might undermine the generalizability of our findings, but it allowed a timely evaluation of risk perception during the pandemic peak while guaranteeing social distancing. Another possible limit concerns the adoption of short -but psychometrically sounded- measures to evaluate complex phenomena, such as the ECR-12 for Attachment Insecurity and the TIPI for the Big Fives personality traits. However, brief measures were chosen to shorten an already length survey, reducing both fatigue and boredom and increasing participants' motivation to respond (Brugnera et al., 2019). Also, we adopted a cross-sectional design, which is inherent to the object of the investigation, but prevents the identification of causal effects among the variables considered (Kazdin, 2021). At least, the statistical approach used weights the possible effect of each variable by the simultaneous effect of all the other variables, which is especially valuable when considering multidimensional and complex phenomena such as risk perception. Finally, this study focused on the Italian population, but risk perception for COVID-19 and its determinants might differ across countries and cultures (De Zwart et al., 2007, 2009; Cho and Lee, 2015).

To conclude, our findings show that risk perception for COVID-19 is a complex phenomenon, and several determinants can be identified including sociodemographic and epidemiological factors, but also psychological variables. Indeed, our findings preliminary show that certain psychological dimensions, such as attachment, personality traits, and locus of control influence the perception of risk, which in turn affects the adoption of preventive behaviors. Nonetheless, the investigation of the psychological determinants of risk perception for infectious respiratory diseases has been quite neglected. Thus, future studies should further investigate this issue, taking into consideration the simultaneous and intricate interplay of multiple variables. Furthermore, our results showed that psychological factors also modulate the adherence to preventive behaviors, not only through their effect on risk perception but also with a direct effect.

Indeed, a comprehensive understanding of the determinants of risk perception is essential to modulate the perceived risk and, consequently, to favor optimal adherence to preventive behaviors. Our results may help policymakers in focusing the available, and usually limited, resources on those targets most likely to have biased risk perceptions. Also, they may contribute to the identification of the dimensions on which to leverage. Overall, our findings suggest that people who easily worry, emotionally overreact, and feel powerless, are more prone to perceive high risk. This possibly favor their engagement in preventive behaviors, at least to a certain extent; however, it is well-known that experiencing too intense fear and psychological distress may be paralyzing and promote dysfunctional behaviors, especially when you feel no control over the situation. Accordingly, these people may benefit of prompt reassurance and adequate psychological support to lower, and manage, the excessive emotional arousal. Also, they may take advantage of clear and easy-to-follow behavioral guidelines, promoting their empowerment, self-confidence and, eventually, adherence. On the contrary, less anxious individuals, who are more emotionally disengaged, reflective and open to—possibly adverse—experience seem to perceive low risk and to be less motivated to engage in precautionary measures. Concrete and plausible examples that *they* can be affected and, possibly, severely harmed might favor a functional risk re-appraisal and proper compliance.

This is true for COVID-19, but this knowledge might also be useful to face possible future pandemics. Therefore, our findings may be taken into high consideration by stakeholders who are responsible for promoting a truthful perception of risk and proper compliance with precautionary measures. Ultimately, better management of similar scenarios might contribute reducing the psychological distress and relational issues associated with infectious outbreaks and quarantine (Brooks et al., 2020; Ferrucci et al., 2020; Panzeri et al., 2020).

## Data Availability Statement

The raw data supporting the conclusions of this article will be made available by the authors, without undue reservation.

## Ethics Statement

The study was reviewed and approved by the Ethics Committee of the IRCCS Istituto Auxologico Italiano. Participants provided their written informed consent to participate in this study.

## Author Contributions

ST: conceptualization, data collection, data curation, results discussion and interpretation, writing—original draft preparation and reviewing. AB: conceptualization, data collection, data curation, formal analysis, writing—original draft preparation, and reviewing. RF: supervision and reviewing of the final draft. KM: supervision and reviewing of the final draft. LP: supervision and reviewing of the final draft. AP: supervision and reviewing of the final draft. NT: supervision and reviewing of the final draft. AC: conceptualization, supervision, and reviewing of the final draft. VS: supervision and reviewing of the final draft. GP: supervision and reviewing of the final draft. BP: conceptualization, supervision, results discussion and interpretation, writing—reviewing. All authors contributed to the article and approved the submitted version.

## Conflict of Interest

The authors declare that the research was conducted in the absence of any commercial or financial relationships that could be construed as a potential conflict of interest.
