# Supplementary material for: Attachment, Personality and Locus of Control: Psychological Determinants of Risk Perception and Preventive Behaviors for COVID-19
Source: Front Psychol. 2021 Jul 9;12:634012. doi: 10.3389/fpsyg.2021.634012 (PMC8299752; doi:10.3389/fpsyg.2021.634012)
Supplement: Supplementary file 1 [file Data_Sheet_1.docx]

**Attachment, Personality and Locus of Control: Psychological Determinants of Risk Perception and Preventive Behaviors for COVID-19**

Sofia Tagini, Agostino Brugnera, Roberta Ferrucci, Ketti Mazzocco, Luca Pievani, Alberto Priori, Nicola Ticozzi, Angelo Compare, Vincenzo Silani, Gabriella Pravettoni and Barbara Poletti

**Supplementary Materials**

**Exploratory Factorial Analysis (EFA)**

As a preliminary step, we performed an Exploratory Factorial Analysis (EFA) on the Risk Perception scale. Both the Kaiser–Meyer–Olkin measure of sampling adequacy (KMO; Kaiser, 1974) and Bartlett's Test of sphericity (Bartlett, 1954) showed the suitability of the correlation matrix for the EFA (KMO = 0.733; Bartlett’s Test = *p* < 0.001). Further, according to their skewness and kurtosis values, all variables were normally distributed (i.e., skewness < 2, kurtosis < 7; West, Finch, & Curran, 1995).

The factorial structure of the scale was assessed through a Principal Component Analysis, with Varimax rotation. All communalities were above .40 (range .67 – .85), suggesting that the variance of all items was adequately captured by the number of factors extracted (Mulaik, 2009). Results showed that only two factors had eigenvalues greater than one, accounting for 76.8% of the variance within the scale items: these results were supported also by a visual analysis of the scree-plot and by the results of the Horn’s parallel analysis. Finally, all loadings were higher than 0.40, with no significant cross-loadings (i.e., > .30 on the second component). The two components were named Severity (3 items) and Vulnerability (2 items). We additionally tested different solutions (i.e., using a Maximum Likelihood extraction method, or adopting an oblique rotation) but results were respectively unchanged, or factors were poorly correlated among them (*r* < .20). Descriptive statistics for all items, total variances explained, and factor loadings are reported in Supplementary Tables 1-3. Finally, both scales had a good internal reliability (α of .89 for Severity; inter-item correlation of .38 for Vulnerability). In accordance with previous literature (De Zwart et al., 2007, 2009), we then computed the product of the Severity factor for the Vulnerability one, obtaining a new scale called “Risk Perception”, which was used in all analyses.

**Supplementary Table 1.**

*Descriptive statistics (mean and standard deviation) for the 5-items of the Risk Perception scale (N = 911).*

| **Items** |  | **Mean** | **SD** | **Range** | **Skewness** | **Kurtosis** |
| --- | --- | --- | --- | --- | --- | --- |
| **Severity Item 1.** ***Being infected by COVID-19 can be a very serious problem*** |  | 4.45 | .78 | 2-5 | -1.241 | .721 |
| **Severity Item 2. *It can be difficult to treat people with COVID-19*** |  | 4.31 | .83 | 1-5 | -1.018 | .592 |
| **Severity Item 3.** ***Contracting COVID-19 can be very dangerous*** |  | 4.34 | .85 | 1-5 | -1.053 | .365 |
| **Vulnerability Item 1. *It is likely that I can* *get COVID-19*** |  | 3.32 | 1.02 | 1-5 | .036 | -.519 |
| **Vulnerability Item 2. *I am more likely to get COVID-19 than other people of my age and sex*** |  | 2.42 | 1.25 | 1-5 | .574 | -.631 |

**Supplementary Table 2.**

*Total variance explained by the EFA on the 5-items of the “Perceived Risk” scale. Components were extracted through a Principal Component Analysis (N = 911).*

| **Total Variance Explained** | | | | | | | | | |
| --- | --- | --- | --- | --- | --- | --- | --- | --- | --- |
| Component | Initial Eigenvalues | | | Extraction Sums of Squared Loadings | | | Rotation Sums of Squared Loadings | | |
|  | Total | % of Variance | Cumulative % | Total | % of Variance | Cumulative % | Total | % of Variance | Cumulative % |
| **1** | 2.605 | 52.104 | 52.104 | 2.605 | 52.104 | 52.104 | 2.444 | 48.885 | 48.885 |
| **2** | 1.232 | 24.647 | 76.751 | 1.232 | 24.647 | 76.751 | 1.393 | 27.866 | 76.751 |
| **3** | .607 | 12.144 | 88.895 |  |  |  |  |  |  |
| **4** | .337 | 6.740 | 95.635 |  |  |  |  |  |  |
| **5** | .218 | 4.365 | 100.000 |  |  |  |  |  |  |

**Supplementary Table 3.**

*Rotated Component Matrix for the EFA on the items of the scale “Risk Perception”. Components were extracted with a Principal Component Analysis, and factors were rotated with a Varimax method (N = 911).*

| **Items** | **Component** | |
| --- | --- | --- |
|  | **1** | **2** |
| **Severity Item 1** | .920 | -.012 |
| **Severity Item 2** | .832 | .024 |
| **Severity Item 3** | .798 | -.010 |
| **Vulnerability Item 1** | .044 | .728 |
| **Vulnerability Item 2** | -.026 | .522 |

**Supplementary Table 4.**

*Zero-order correlations among all sociodemographic, epidemiological, and psychological variables examined in this study.*

| **Variables** |  | **1.** | **2.** | **3.** | **4.** | **5.** | **6.** | **7.** | **8.** | **9.** | **10.** | **11.** | **12.** | **13.** | **14.** | **15.** | **16.** |
| --- | --- | --- | --- | --- | --- | --- | --- | --- | --- | --- | --- | --- | --- | --- | --- | --- | --- |
| **1. Risk Perception** | *r* | \ |  |  |  |  |  |  |  |  |  |  |  |  |  |  |  |
|  | *N* |  |  |  |  |  |  |  |  |  |  |  |  |  |  |  |  |
| **2. Age** | *r* | .015 | \ |  |  |  |  |  |  |  |  |  |  |  |  |  |  |
|  | *N* | 911 | 911 |  |  |  |  |  |  |  |  |  |  |  |  |  |  |
| **3. Sex - reference category Men** | *r* | -.080^*^ | -.006 | \ |  |  |  |  |  |  |  |  |  |  |  |  |  |
|  | *N* | 911 | 911 |  |  |  |  |  |  |  |  |  |  |  |  |  |  |
| **4. Filling out the battery during Lockdown** | *r* | .003 | .059 | -.150^**^ | \ |  |  |  |  |  |  |  |  |  |  |  |  |
|  | *N* | 911 | 911 | 911 |  |  |  |  |  |  |  |  |  |  |  |  |  |
| **5. Living in area with more than 1% of infected Population** | *r* | .030 | -.248^**^ | -.001 | -.291^**^ | \ |  |  |  |  |  |  |  |  |  |  |  |
|  | *N* | 875 | 875 | 875 | 875 |  |  |  |  |  |  |  |  |  |  |  |  |
| **6. Education (University)** | *r* | .025 | -.222^**^ | -.053 | .038 | -.043 | \ |  |  |  |  |  |  |  |  |  |  |
|  | *N* | 911 | 911 | 911 | 911 | 875 |  |  |  |  |  |  |  |  |  |  |  |
| **7. Civil Status (Married/in a Relationship)** | *r* | -.009 | .007 | .012 | -.052 | .099^**^ | .039 | \ |  |  |  |  |  |  |  |  |  |
|  | *N* | 911 | 911 | 911 | 911 | 875 | 911 |  |  |  |  |  |  |  |  |  |  |
| **8. Living with people at High Risk** | *r* | .099^**^ | .000 | -.032 | .032 | .064 | -.082^*^ | -.044 | \ |  |  |  |  |  |  |  |  |
|  | *N* | 900 | 900 | 900 | 900 | 864 | 900 | 900 |  |  |  |  |  |  |  |  |  |
| **9. Physical Health** | *r* | -.120^**^ | -.195^**^ | .095^**^ | -.019 | -.030 | .064 | -.039 | -.008 | \ |  |  |  |  |  |  |  |
|  | *N* | 911 | 911 | 911 | 911 | 875 | 911 | 911 | 900 |  |  |  |  |  |  |  |  |
| **10. Chronic Diseases** | *r* | .083^*^ | .306^**^ | -.095^**^ | .092^**^ | -.058 | -.176^**^ | .021 | .042 | -.375^**^ | \ |  |  |  |  |  |  |
|  | *N* | 911 | 911 | 911 | 911 | 875 | 911 | 911 | 900 | 911 |  |  |  |  |  |  |  |
| **11. COVID-like symptoms** | *r* | .114^**^ | -.045 | -.030 | -.067^*^ | .244^**^ | -.020 | .009 | .061 | -.022 | -.040 | \ |  |  |  |  |  |
|  | *N* | 911 | 911 | 911 | 911 | 875 | 911 | 911 | 900 | 911 | 911 |  |  |  |  |  |  |
| **12. COVID-19 diagnosis** | *r* | .022 | .025 | .024 | -.003 | .061 | .054 | -.015 | -.001 | -.031 | .000 | .155^**^ | \ |  |  |  |  |
|  | *N* | 911 | 911 | 911 | 911 | 875 | 911 | 911 | 900 | 911 | 911 | 911 |  |  |  |  |  |
| **13. Cases among close friends or relatives** | *r* | .066^*^ | -.051 | -.089^**^ | .007 | .276^**^ | -.076^*^ | .054 | -.010 | -.041 | .031 | .143^**^ | -.017 | \ |  |  |  |
|  | *N* | 911 | 911 | 911 | 911 | 875 | 911 | 911 | 900 | 911 | 911 | 911 | 911 |  |  |  |  |
| **14. Deaths among close friends or relatives** | *r* | .126^**^ | .013 | -.045 | -.032 | .259^**^ | -.094^**^ | .032 | .068^*^ | .009 | .063 | .112^**^ | .001 | .453^**^ | \ |  |  |
|  | *N* | 911 | 911 | 911 | 911 | 875 | 911 | 911 | 900 | 911 | 911 | 911 | 911 | 911 |  |  |  |
| **15. Working near\in contact with COVID-19 patients** | *r* | .230^**^ | -.046 | -.018 | -.008 | .085^*^ | .120^**^ | .054 | .021 | .051 | -.068^*^ | .154^**^ | .139^**^ | .108^**^ | .054 | \ |  |
|  | *N* | 911 | 911 | 911 | 911 | 875 | 911 | 911 | 900 | 911 | 911 | 911 | 911 | 911 | 911 |  |  |
| **16. Adequacy of received Info** | *r* | .063 | .009 | -.006 | -.087^**^ | -.047 | -.012 | -.004 | -.007 | .090^**^ | -.045 | -.037 | .026 | -.029 | -.043 | .056 | \ |
|  | *N* | 911 | 911 | 911 | 911 | 875 | 911 | 911 | 900 | 911 | 911 | 911 | 911 | 911 | 911 | 911 |  |
| **17. Trust in Institutions** | *r* | .047 | .035 | -.010 | -.092^**^ | -.100^**^ | .029 | -.016 | .005 | .105^**^ | -.020 | -.028 | -.044 | -.094^**^ | -.094^**^ | -.006 | .440^**^ |
|  | *N* | 911 | 911 | 911 | 911 | 875 | 911 | 911 | 900 | 911 | 911 | 911 | 911 | 911 | 911 | 911 | 911 |
| **18. Adoption of Preventive Measures** | *r* | .178^**^ | .140^**^ | -.139^**^ | -.180^**^ | .049 | -.061 | -.017 | .028 | -.044 | .066^*^ | .083^*^ | .005 | .063 | .047 | .027 | .172^**^ |
|  | *N* | 911 | 911 | 911 | 911 | 875 | 911 | 911 | 900 | 911 | 911 | 911 | 911 | 911 | 911 | 911 | 911 |
| **19. ECR-12 Anxiety** | *r* | .176^**^ | -.188^**^ | -.068^*^ | .000 | .033 | .020 | -.166^**^ | .035 | -.041 | -.062 | .063 | -.037 | .046 | .019 | -.003 | -.046 |
|  | *N* | 911 | 911 | 911 | 911 | 875 | 911 | 911 | 900 | 911 | 911 | 911 | 911 | 911 | 911 | 911 | 911 |
| **20. ECR-12 Avoidance** | *r* | -.039 | .214^**^ | .066^*^ | .044 | -.040 | -.109^**^ | -.213^**^ | -.001 | -.145^**^ | .128^**^ | -.054 | -.044 | -.068^*^ | .011 | -.072^*^ | -.103^**^ |
|  | *N* | 911 | 911 | 911 | 911 | 875 | 911 | 911 | 900 | 911 | 911 | 911 | 911 | 911 | 911 | 911 | 911 |
| **21. PHQ-9** | *r* | .186^**^ | -.158^**^ | -.140^**^ | -.038 | .026 | -.006 | -.018 | .082^*^ | -.311^**^ | .075^*^ | .134^**^ | .023 | .101^**^ | .028 | -.044 | -.118^**^ |
|  | *N* | 911 | 911 | 911 | 911 | 875 | 911 | 911 | 900 | 911 | 911 | 911 | 911 | 911 | 911 | 911 | 911 |
| **22. GAD-7** | *r* | .219^**^ | -.137^**^ | -.172^**^ | -.001 | .041 | -.043 | .026 | .102^**^ | -.229^**^ | .051 | .105^**^ | -.008 | .108^**^ | .095^**^ | -.062 | -.099^**^ |
|  | *N* | 911 | 911 | 911 | 911 | 875 | 911 | 911 | 900 | 911 | 911 | 911 | 911 | 911 | 911 | 911 | 911 |
| **23. GSE** | *r* | -.060 | .090^**^ | .038 | -.039 | -.038 | .073^*^ | -.020 | -.026 | .177^**^ | -.026 | -.001 | .032 | .000 | .009 | .113^**^ | .155^**^ |
|  | *N* | 911 | 911 | 911 | 911 | 875 | 911 | 911 | 900 | 911 | 911 | 911 | 911 | 911 | 911 | 911 | 911 |
| **24. TIPI Extroversion** | *r* | -.019 | .094^**^ | -.020 | -.032 | -.026 | .010 | .076^*^ | -.042 | .099^**^ | -.016 | .042 | .040 | .010 | .020 | .024 | .074^*^ |
|  | *N* | 911 | 911 | 911 | 911 | 875 | 911 | 911 | 900 | 911 | 911 | 911 | 911 | 911 | 911 | 911 | 911 |
| **25. TIPI Agreeableness** | *r* | .039 | .065^*^ | -.141^**^ | .012 | -.019 | .027 | -.050 | .003 | .063 | .010 | .001 | .077^*^ | .007 | -.015 | .046 | .137^**^ |
|  | *N* | 911 | 911 | 911 | 911 | 875 | 911 | 911 | 900 | 911 | 911 | 911 | 911 | 911 | 911 | 911 | 911 |
| **26. TIPI Conscientiousness** | *r* | .005 | .038 | -.104^**^ | .060 | -.040 | .036 | .003 | .002 | .101^**^ | .023 | -.007 | .006 | -.027 | -.012 | -.003 | .050 |
|  | *N* | 911 | 911 | 911 | 911 | 875 | 911 | 911 | 900 | 911 | 911 | 911 | 911 | 911 | 911 | 911 | 911 |
| **27. TIPI Emotional Stability** | *r* | -.087^**^ | .199^**^ | .124^**^ | -.019 | -.054 | .015 | -.047 | -.071^*^ | .189^**^ | -.008 | -.066^*^ | .052 | -.037 | -.045 | .102^**^ | .129^**^ |
|  | *N* | 911 | 911 | 911 | 911 | 875 | 911 | 911 | 900 | 911 | 911 | 911 | 911 | 911 | 911 | 911 | 911 |
| **28. TIPI Openness** | *r* | -.080^*^ | .089^**^ | -.038 | .019 | -.035 | .064 | -.031 | -.026 | .075^*^ | .046 | .056 | .054 | .041 | .043 | .074^*^ | .017 |
|  | *N* | 911 | 911 | 911 | 911 | 875 | 911 | 911 | 900 | 911 | 911 | 911 | 911 | 911 | 911 | 911 | 911 |
| **29. Brief-COPE Approach** | *r* | .055 | -.080^*^ | -.247^**^ | .070^*^ | -.002 | .136^**^ | -.021 | .036 | .065^*^ | -.067^*^ | .038 | .038 | .090^**^ | .041 | .068^*^ | .061 |
|  | *N* | 911 | 911 | 911 | 911 | 875 | 911 | 911 | 900 | 911 | 911 | 911 | 911 | 911 | 911 | 911 | 911 |
| **30. Brief-COPE Avoidant** | *r* | .163^**^ | -.066^*^ | -.142^**^ | -.004 | -.015 | -.008 | -.100^**^ | .028 | -.034 | -.041 | .022 | -.042 | .063 | .056 | -.029 | .000 |
|  | *N* | 911 | 911 | 911 | 911 | 875 | 911 | 911 | 900 | 911 | 911 | 911 | 911 | 911 | 911 | 911 | 911 |
| **31. H-LoC Internal** | *r* | .012 | .005 | .073^*^ | -.050 | -.025 | -.094^**^ | .032 | -.011 | .105^**^ | -.024 | .014 | .041 | -.030 | -.027 | -.007 | .032 |
|  | *N* | 911 | 911 | 911 | 911 | 875 | 911 | 911 | 900 | 911 | 911 | 911 | 911 | 911 | 911 | 911 | 911 |
| **32. H-LoC External God** | *r* | .103^**^ | .170^**^ | -.076^*^ | .084^*^ | -.023 | -.058 | .068^*^ | .030 | -.049 | .035 | .027 | -.016 | .045 | .117^**^ | -.056 | -.033 |
|  | *N* | 911 | 911 | 911 | 911 | 875 | 911 | 911 | 900 | 911 | 911 | 911 | 911 | 911 | 911 | 911 | 911 |
| **33. H-LoC External Others** | *r* | .138^**^ | -.133^**^ | .059 | -.020 | .043 | -.001 | -.011 | .068^*^ | -.036 | -.037 | .019 | -.027 | .023 | .056 | .007 | .006 |
|  | *N* | 911 | 911 | 911 | 911 | 875 | 911 | 911 | 900 | 911 | 911 | 911 | 911 | 911 | 911 | 911 | 911 |

**Supplementary Table 4 (continues).**

*Zero-order correlations among all sociodemographic, epidemiological, and psychological variables examined in this study.*

| **Variables** |  | **17.** | **18.** | **19.** | **20.** | **21.** | **22.** | **23.** | **24.** | **25.** | **26.** | **27.** | **28.** | **29.** | **30.** | **31.** | **32.** |
| --- | --- | --- | --- | --- | --- | --- | --- | --- | --- | --- | --- | --- | --- | --- | --- | --- | --- |
| **18. Adoption of Preventive Measures** | *r* | .205^**^ | \ |  |  |  |  |  |  |  |  |  |  |  |  |  |  |
|  | *N* | 911 |  |  |  |  |  |  |  |  |  |  |  |  |  |  |  |
| **19. ECR-12 Anxiety** | *r* | -.007 | -.038 | \ |  |  |  |  |  |  |  |  |  |  |  |  |  |
|  | *N* | 911 | 911 |  |  |  |  |  |  |  |  |  |  |  |  |  |  |
| **20. ECR-12 Avoidance** | *r* | -.056 | -.095^**^ | .030 | \ |  |  |  |  |  |  |  |  |  |  |  |  |
|  | *N* | 911 | 911 | 911 |  |  |  |  |  |  |  |  |  |  |  |  |  |
| **21. PHQ-9** | *r* | -.118^**^ | .031 | .290^**^ | .133^**^ | \ |  |  |  |  |  |  |  |  |  |  |  |
|  | *N* | 911 | 911 | 911 | 911 |  |  |  |  |  |  |  |  |  |  |  |  |
| **22. GAD-7** | *r* | -.119^**^ | .050 | .274^**^ | .091^**^ | .770^**^ | \ |  |  |  |  |  |  |  |  |  |  |
|  | *N* | 911 | 911 | 911 | 911 | 911 |  |  |  |  |  |  |  |  |  |  |  |
| **23. GSE** | *r* | .151^**^ | .075^*^ | -.217^**^ | -.187^**^ | -.357^**^ | -.350^**^ | \ |  |  |  |  |  |  |  |  |  |
|  | *N* | 911 | 911 | 911 | 911 | 911 | 911 |  |  |  |  |  |  |  |  |  |  |
| **24. TIPI Extroversion** | *r* | .049 | -.059 | -.076^*^ | -.150^**^ | -.114^**^ | -.114^**^ | .258^**^ | \ |  |  |  |  |  |  |  |  |
|  | *N* | 911 | 911 | 911 | 911 | 911 | 911 | 911 |  |  |  |  |  |  |  |  |  |
| **25. TIPI Agreeableness** | *r* | .164^**^ | .194^**^ | -.105^**^ | -.159^**^ | -.162^**^ | -.154^**^ | .170^**^ | -.014 | \ |  |  |  |  |  |  |  |
|  | *N* | 911 | 911 | 911 | 911 | 911 | 911 | 911 | 911 |  |  |  |  |  |  |  |  |
| **26. TIPI Conscientiousness** | *r* | .073^*^ | .105^**^ | -.177^**^ | -.151^**^ | -.243^**^ | -.139^**^ | .283^**^ | -.013 | .160^**^ | \ |  |  |  |  |  |  |
|  | *N* | 911 | 911 | 911 | 911 | 911 | 911 | 911 | 911 | 911 |  |  |  |  |  |  |  |
| **27. TIPI Emotional Stability** | *r* | .120^**^ | .022 | -.323^**^ | -.092^**^ | -.477^**^ | -.533^**^ | .479^**^ | .081^*^ | .296^**^ | .223^**^ | \ |  |  |  |  |  |
|  | *N* | 911 | 911 | 911 | 911 | 911 | 911 | 911 | 911 | 911 | 911 |  |  |  |  |  |  |
| **28. TIPI Openness** | *r* | -.003 | .039 | -.088^**^ | -.101^**^ | -.055 | -.086^**^ | .287^**^ | .309^**^ | .141^**^ | -.014 | .157^**^ | \ |  |  |  |  |
|  | *N* | 911 | 911 | 911 | 911 | 911 | 911 | 911 | 911 | 911 | 911 | 911 |  |  |  |  |  |
| **29. Brief-COPE Approach** | *r* | .039 | .094^**^ | .059 | -.253^**^ | .007 | .016 | .214^**^ | .104^**^ | .197^**^ | .096^**^ | .025 | .197^**^ | \ |  |  |  |
|  | *N* | 911 | 911 | 911 | 911 | 911 | 911 | 911 | 911 | 911 | 911 | 911 | 911 |  |  |  |  |
| **30. Brief-COPE Avoidant** | *r* | -.013 | .008 | .232^**^ | .010 | .312^**^ | .362^**^ | -.116^**^ | -.001 | .019 | -.086^**^ | -.220^**^ | .001 | -.386^**^ | \ |  |  |
|  | *N* | 911 | 911 | 911 | 911 | 911 | 911 | 911 | 911 | 911 | 911 | 911 | 911 | 911 |  |  |  |
| **31 H-LoC Internal** | *r* | .109^**^ | .015 | -.043 | -.082^*^ | -.082^*^ | -.053 | .223^**^ | .026 | .059 | .067^*^ | .124^**^ | .043 | .079^*^ | -.039 | \ |  |
|  | *N* | 911 | 911 | 911 | 911 | 911 | 911 | 911 | 911 | 911 | 911 | 911 | 911 | 911 | 911 |  |  |
| **32. H-LoC External God** | *r* | -.015 | .028 | .062 | .008 | -.007 | .099^**^ | -.039 | -.001 | .059 | .050 | -.025 | -.120^**^ | .028 | .252^**^ | -.105^**^ | \ |
|  | *N* | 911 | 911 | 911 | 911 | 911 | 911 | 911 | 911 | 911 | 911 | 911 | 911 | 911 | 911 | 911 |  |
| **33. H-LoC External Others** | *r* | -.040 | -.084^*^ | .205^**^ | .031 | .231^**^ | .224^**^ | -.164^**^ | -.003 | -.121^**^ | -.133^**^ | -.163^**^ | -.037 | -.066^*^ | .194^**^ | -.219^**^ | .155^**^ |
|  | *N* | 911 | 911 | 911 | 911 | 911 | 911 | 911 | 911 | 911 | 911 | 911 | 911 | 911 | 911 | 911 | 911 |

*Note.* * = correlation is significant at the .05 level; ** = correlation is significant at the .01 level. ECR-12 = Experiences in Close Relationships - 12; PHQ-9 = Patient Health Questionnaire - 9; GAD-7 = General Anxiety Disorder Scale - 7; GSE = General Self-Efficacy scale; TIPI = Ten-Item Personality Inventory; Brief-COPE = Brief - Coping Orientation to Problems Experienced; H-LoC = Health-related Locus of Control scale.

**Supplementary Table 5.**

*Unstandardized Regression Weights, Standard Errors, Beta, t- and p-values for the Direct Effects of all variables in the model where Perceived Risk mediate the association between sociodemographic, epidemiological and psychological factors and the adoption of preventive measures (N = 907).*

| **Predictors** | **Predicted Variables** | **B** | **Beta** | **SE** | ***t*-value** | ***p*-value** |
| --- | --- | --- | --- | --- | --- | --- |
| Deaths among close friends or relatives | Risk Perception | 0.413 | .099 | 0.13 | 3.191 | .001 |
| Working near\in contact with COVID patients | Risk Perception | 1.264 | .232 | 0.169 | 7.475 | < .001 |
| H-LoC External Others | Risk Perception | 1.33 | .065 | 0.653 | 2.036 | .042 |
| TIPI Openness | Risk Perception | -0.116 | -.078 | 0.047 | -2.487 | .013 |
| GAD-7 | Risk Perception | 0.39 | .181 | 0.071 | 5.502 | < .001 |
| ECR-12 Anxiety | Risk Perception | 0.42 | .102 | 0.134 | 3.133 | .002 |
| ECR-12 Avoidance | Risk Perception | -0.266 | -.036 | 0.234 | -1.134 | .257 |
| Adequacy of received info | Risk Perception | 0.036 | .059 | 0.019 | 1.887 | .059 |
| Risk Perception | Adoption of Preventive Measures | 0.02 | .196 | 0.003 | 5.755 | < .001 |
| Deaths among close friends or relatives | Adoption of Preventive Measures | 0.011 | .027 | 0.013 | 0.85 | .395 |
| Working near\in contact with COVID patients | Adoption of Preventive Measures | -0.014 | -.026 | 0.018 | -0.799 | .425 |
| H-LoC External Others | Adoption of Preventive Measures | -0.249 | -.121 | 0.068 | -3.68 | < .001 |
| TIPI Openness | Adoption of Preventive Measures | 0.008 | .053 | 0.005 | 1.639 | .101 |
| GAD-7 | Adoption of Preventive Measures | 0.014 | .065 | 0.007 | 1.908 | .056 |
| ECR-12 Anxiety | Adoption of Preventive Measures | -0.022 | -.052 | 0.014 | -1.561 | .119 |
| ECR-12 Avoidance | Adoption of Preventive Measures | -0.053 | -.069 | 0.024 | -2.164 | .030 |
| Adequacy of received info | Adoption of Preventive Measures | 0.011 | .171 | 0.002 | 5.341 | < .001 |

*Note.* ECR-12 = Experiences in Close Relationships - 12; GAD-7 = General Anxiety Disorder Scale - 7; TIPI = Ten-Item Personality Inventory; H-LoC = Health-related Locus of Control scale.

**Supplementary Table 6.**

*Standardized Indirect Effects, 95% bootstrapped CIs and their significance level. If significant, then the specific predictor asserts an indirect effect on the Adoption on Preventive Measures through Risk Perception.*

| **Predictors** | **Standardized Indirect Effect** | **95% CIs LB** | **95% CI UB** | ***p*-value** |
| --- | --- | --- | --- | --- |
| Adequacy of received info | .012 | .028 | -.001 | .057 |
| ECR-12 Avoidance | -.007 | .005 | -.021 | .237 |
| H-LoC External Others | .013 | .001 | .029 | .039 |
| TIPI Openness | -.015 | -.004 | -.03 | .008 |
| GAD-7 | .035 | .057 | .02 | < .001 |
| ECR-12 Anxiety | .020 | .037 | .007 | .003 |
| Working near\in contact with COVID patients | .046 | .069 | .027 | < .001 |
| Deaths among close friends or relatives | .019 | .036 | .007 | .001 |

*Note.* ECR-12 = Experiences in Close Relationships - 12; GAD-7 = General Anxiety Disorder Scale - 7; TIPI = Ten-Item Personality Inventory; H-LoC = Health-related Locus of Control scale. CIs = Confidence Intervals (LB = Lower Bound; UB = Upper Bound).
